# Surgical management of obturator neuropathy with a concomitant acetabular labral tear — a case report

**DOI:** 10.1080/17453674.2018.1494118

**Published:** 2018-07-09

**Authors:** Shiho Kanezaki, Akinori Sakai, Eiichiro Nakamura, Soshi Uchida

**Affiliations:** 1 Department of Orthopedic Surgery, Wakamatsu Hospital for the University of Occupational and Environmental Health;; 2 Department of Orthopedic Surgery, University of Occupational and Environmental Health, Fukuoka, Japan

A 42-year-old woman presented to our hospital with a 3-month history of right groin pain after an accident. While cycling she collided with a car, which hit her frontally on the right side. She was immediately transferred to the hospital. Pain did not improve with NSAIDs and was becoming progressively worse. She was unable to perform deep squats, walk for more than10 minutes or climb stairs. The pain was located in the anterior and medial aspect of the right groin.

The patient had limited hip motion with 80° of flexion and 20° of abduction. With the hip in 90° flexion to the supine position, the patient had 5° of external rotation and 10° of internal rotation. Pain was reproduced on flexion adduction and internal rotation (FADIR) test, a positive impingement test. The hip dial test was positive.

Manual muscle strength (MMT) of the adductor muscles of the right hip was fair (3/5). Strengths of other hip muscles were normal.

An anterior-posterior (AP) pelvis and Dunn radiological views showed a lateral center edge (LCE) angle of 33°, an alpha angle of 69°, an offset ratio of 0.09, acetabular roof angle of 8° and absence of the crossover sign (an indicator of Cam type femoroacetabular impingement [FAI]). No other major abnormalities were observed. A 3D CT showed that the femoral version was 27° and the acetabular version was 22°. MRI confirmed an antero-superior labral tear and the presence of a Cam lesion (Figure 1A). The patient was diagnosed with Cam-type FAI and concomitant labral tear. Since her pain was refractory to rest, NSAIDs, 3 months of physiotherapy, and a trial of 5 mL of 1% lidocaine injection into the hip joint, hip arthroscopy surgery was performed. A tear of the anterosuperior labrum was confirmed (Figure 1B) and repaired using 3 suture anchors (Gryphon BR, DePuy Synthes, Monument, CO, USA) (Figure 1C).

**Figure 1.**
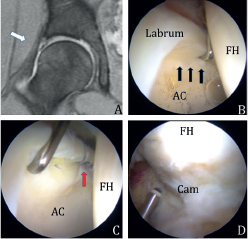
Coronal view of the affected hip and arthroscopic finding showing an antero-superior acetabular labral tear. (A) T2-star coronal view showing the high intensity of an acetabular labral tear. (B) Arthroscopic findings from an anterolateral portal revealed an antero-superior labral tear. (C) Labral repair with suture anchors. (D) View of Cam impingement. *FH: femoral head. AC: acetabulum.

After releasing traction, a dynamic impingement test confirmed significant impingement on abduction and flexion. Osteochondroplasty was then performed (Figure 1D). After osteochondroplasty, an intraoperative dynamic impingement test confirmed removal of the impingement and an effectively sealed labrum. Capsular repair was performed as described previously (Murata et al. 2017). The patient completed a rehabilitation protocol and full weight-bearing was allowed at 5 weeks after surgery. Postoperative Dunn radiological views showed an alpha angle of 45°, and an offset ratio of 0.18.

At 4 months postoperatively, the patient began to complain of medial thigh paresthesia and at 12 months postoperatively she reported improved range of motion (ROM) but continued discomfort with the same medial groin pain as before arthroscopy. Pain was localized to the inner side of the thigh over the adductor muscle, starting 2 cm distal to the inguinal ligament (Figure 2A). An MRI scan was performed, confirming appropriate healing of the labrum without evidence of any abnormality. There was nothing changed on this MRI from the preoperative MRI in the obturator nerve or magnus area. MMT of the affected hip adductor was still fair (3/5).

**Figure 2.**
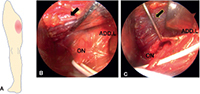
Image of obturator nerve pain and operative findings during obturator nerve release. (A) Patients complained of medial thigh pain and numbness. (B) The anterior branch of the obturator nerve was identified over the adductor brevis. Neurolysis of the obturator nerve (ON) was performed. View of adductor longus (ADD.L); arrow pointing to the overlying fascia. (C) Note the nerve being trapped by overlying fascia. Arrow: overlying facia.

An obturator neuropathy was suspected. Although electromyography (EMG) showed no evidence of denervation in the short and long adductor muscles, we suspected an obturator nerve entrapment. Using ultrasound, we injected 5 mL of 1% lidocaine and 3.1 mL of corticosteroid into the interfascial space between the adjacent pectineus and adductor brevis muscles (Anagnostopoulou et al. 2008). The patient reported a temporary improvement in her hip pain confirming the diagnosis of obturator nerve entrapment. An open decompression of the obturator nerve was performed via an anterior approach according to Bradshow et al. (1997). The nerve was strangulated by the overlying adhesive fascia (Figure 2B–C).

The patient was allowed to fully weight-bear immediately after surgery. The groin pain improved. At 3 months postoperatively, the hip ROM was improved, with flexion of 120°, abduction of 30°, internal rotation of 40°, and external rotation of 45°. The patient returned to work and became pain-free.

## Discussion

We report a case of obturator neuropathy that presented with groin pain similar to that of FAI. Following the arthroscopic procedure, her clinical hip scores including ROM improved. However, the patient continued to complain of medial groin pain and paresthesia. The persistent medial groin pain was likely resulting from an obturator entrapment that was initially masked by her FAI symptoms. Therefore, when her FAI was addressed, her symptoms did not resolve completely.

Some studies have reported the use of electromyography (EMG) in patients with obturator nerve entrapment. The study by Bradshaw et al. (1997) on athletes with chronic pain secondary to obturator nerve neuropathy reported EMG findings indicative of nerve denervation. Sorenson et al. (2002) and Rigaud et al. (2007) have also described positive EMG findings, which are important in diagnosing obturator nerve neuropathy. However, obturator nerve entrapment can present with normal findings on EMG (Rigaud et al. 2008) and in our case, EMG was normal.

Generally, first-line treatment for obturator neuropathy is non-operative (Tipton 2008) If non-operative modalities fail, surgical intervention with decompression of the obturator nerve may be indicated. Bradshow et al. (1997) have shown that surgically releasing obturator nerve entrapment was effective in 32 athletes with persistent groin pain.

Several studies have shown obturator neuropathy as a potential complication associated with gynecological, urological, and orthopedic surgeries (DeHart and Riley 1999, Hakoiwa et al. 2011). Obturator neuropathy has not been reported as one of the complications after hip arthroscopy (Harris et al. 2013, Kowalczuk et al. 2013). However, we have experienced obturator neuropathy after hip arthroscopic procedures (data not published). Hip traction during the arthroscopy in our case could have worsened the obturator nerve dysfunction.

In summary, surgeons should be mindful that obturator nerve entrapment can present as a source of groin pain and mimic FAI clinically.

*Acta* thanks Hal Martin for help with peer review of this study.
